# Therapeutic effect of erythroid differentiation regulator 1 (Erdr1) on collagen-induced arthritis in DBA/1J mouse

**DOI:** 10.18632/oncotarget.13047

**Published:** 2016-11-03

**Authors:** Kyung Eun Kim, Sungryung Kim, Sunyoung Park, Younkyung Houh, Yoolhee Yang, Seung Beom Park, Sangyoon Kim, Daejin Kim, Dae Young Hur, Seonghan Kim, Hyun Jeong Park, Sa Ik Bang, Daeho Cho

**Affiliations:** ^1^ Department of Cosmetic Sciences, Sookmyung Women's University, Chungpa-Dong 2-Ka, Yongsan-ku, Seoul, Republic of Korea; ^2^ Department of Biological Sciences, Sookmyung Women's University, Chungpa-Dong 2-Ka, Yongsan-ku, Seoul, Republic of Korea; ^3^ Department of Plastic Surgery, Samsung Medical Center, Sungkyunkwan University School of Medicine, 50 Ilwon-dong, Gangnam-gu, Seoul, Republic of Korea; ^4^ Biotech. Team, Cent'l Res. Inst. Ilyang Pharm. Co., Ltd., Gyeonggi-do, Republic of Korea; ^5^ Department of Anatomy, Inje University College of Medicine, Busan, Republic of Korea; ^6^ Department of Dermatology, Yeouido St. Mary's Hospital, The Catholic University of Korea, Seoul, Republic of Korea

**Keywords:** erythroid differentiation regulator 1 (Erdr1), rheumatoid arthritis, inflammation, interleukin-18 (IL-18), synovial fibroblast migration, Immunology and Microbiology Section, Immune response, Immunity

## Abstract

Rheumatoid arthritis (RA) is a chronic inflammatory autoimmune disease, and multiple inflammatory cytokines are involved in RA pathogenesis. Interleukin (IL)-18, in particular, has a significant positive correlation with RA. In this study, we investigated the effect of erythroid differentiation regulator 1 (Erdr1), which is negatively regulated by IL-18, in an animal model of inflammatory arthritis, collagen-induced arthritis (CIA) in DBA/1J mice. Treatment of mice with recombinant (r)Erdr1 significantly suppressed the severity of arthritis, histologic features of arthritic tissue, and serum levels of anti-collagen autoantibodies (IgG, IgG1, IgG2a and IgM) in CIA. In addition, IL-18 expression was reduced in the affected synovium of rErdr1-treated mice. Interestingly, Erdr1 treatment suppressed migration in contrast to the pro-migratory effect of IL-18, indicating the therapeutic effects of Erdr1 on CIA through inhibiting synovial fibroblast migration. In addition, Erdr1 inhibited activation of ERK1/2, a key signaling pathway in migration of various cell types. Taken together, these data show that rErdr1 exerts therapeutic effects on RA by inhibiting synovial fibroblast migration, suggesting that rErdr1 treatment might be an effective therapeutic approach for RA.

## INTRODUCTION

Rheumatoid arthritis (RA) is chronic autoimmune disease that is accompanied by an inflammatory response in the swollen joint, resulting in bone destruction. Although systematic research has not yet elucidated mechanisms and factors underlying RA, it is known that multiple immune cells and pro-inflammatory cytokines are closely related to RA development and progression. RA pathogenesis is a complex inflammatory process caused by various pro-inflammatory cytokines. Interleukin (IL)-12, IL-17, IL-23, and tumor necrosis factor-α (TNF-α) are representative pro-inflammatory cytokines thatare positively correlated with RA severity [1, 2]. Several blocking agents targeting inflammatory cytokines, such as IL-6 and IL-17, have been developed for RA therapy, suggesting key roles of inflammatory cytokines in RA treatment [3, 4]. Notably, many studies show that IL-18, a representative pro-inflammatory cytokine, plays an important role in RA pathogenesis. It has been reported that IL-18 is significantly increased in synovial fluid, synovial tissue, and serum from RA patients, and IL-18 serum level is positively correlated with RA severity [5]. These reports indicate that IL-18 plays a key role in RA pathogenesis.

A previous study reported that erythroid differentiation regulator (Erdr1) is negatively regulated by IL-18 in human and mouse skin tissues [6]. Erdr1, expressed in various normal mouse tissues, was first discovered in the WEHI-3 mouse leukemia cell line and modulates cell growth and survival under diverse stressful conditions. A high concentration of Erdr1 has an inhibitory effect on growth of the BL-70 Burkitt lymphoma cell line, suggesting that Erdr1 regulates the homeostasis of cell growth [7]. Recently, the pro-apoptotic property of Erdr1 was confirmed by the demonstration that rErdr1 induces apoptosis of melanoma cells *via* modulation of apoptosis-regulating factors, such as Bcl-2 and Bax [8]. In addition, recent studies suggest the anti-inflammatory property of Erdr1 in contrast to the pro-inflammatory effects of IL-18. Treatment with rErdr1 has a therapeutic effect on rosacea, an inflammatory skin disease, *via* inhibition of angiogenesis and inflammatory cell infiltration [9]. In addition to improving rosacea, rErdr1 inhibits TNF-α production, inflammatory cell infiltration into lesional skin, and chemokine production in a representative inflammatory skin disease, psoriasis, further supporting an anti-inflammatory function of Erdr1 [6].

Based on our previous studies, we hypothesized that Erdr1 might be part of a therapeutic approach to RA, a representative chronic inflammatory disease. In the present study, we investigated the effect of Erdr1 on RA development and progression using a mouse model of collagen-induced arthritis (CIA). We confirmed that Erdr1 not only alleviated characteristic features of RA, but attenuated pathogenesis of RA by reducing serum levels of anti-collagen- immunoglobulins, downregulating IL-18 expression in synovial tissue, and the functions of synovial fibroblasts, whereby Erdr1 might have a potential therapeutic effect on RA.

## RESULTS

### rErdr1 shows therapeutic effects on CIA *in vivo*

**Figure 1:**
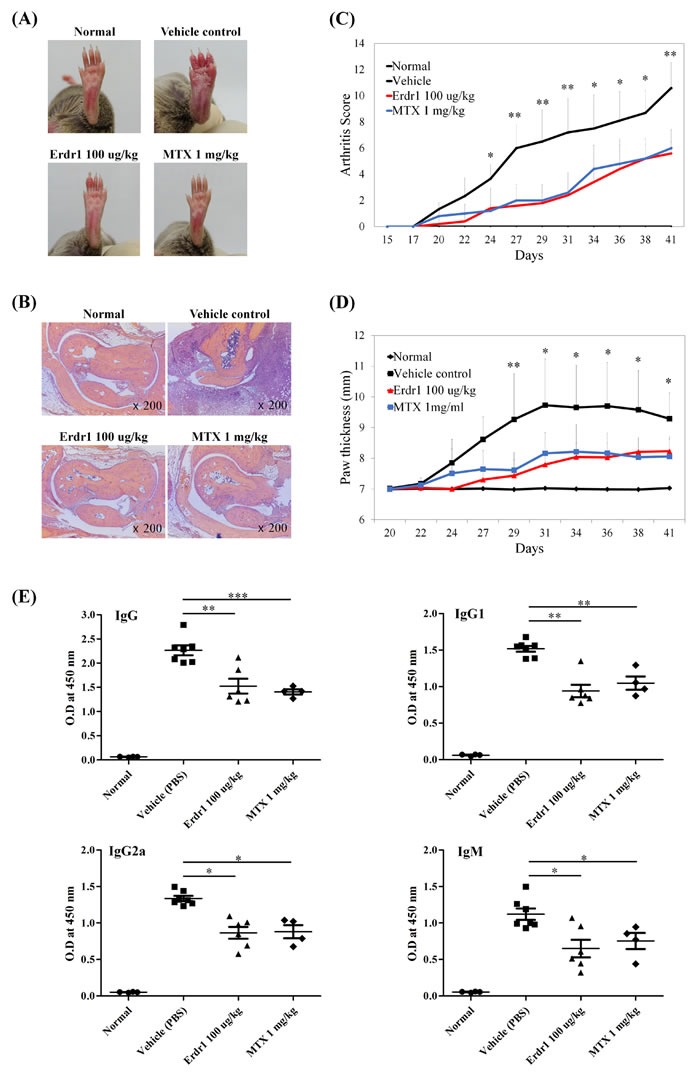
Erdr1 has therapeutic effects on collagen-induced arthritis (CIA) *in vivo* DBA/1J mice were divided into four groups of 8 mice each and immunized with type II collagen (CII), with the exception of the negative control (normal) group. CIA-induced vehicle control group was treated with vehicle PBS after CIA induction. Erdr1-treated group was administered 100 μg/kg rErdr1 three times/week by intraperitoneal (i.p.) injection. MTX was used as a positive control for treatment of CIA. **A.** Gross observation of hind paw. The CIA control showed extremely swollen paw with redness, whereas the Erdr1-treated group showed mild redness and swelling. Photographs are representative of each group. **B.** Histological analysis was performed after H&E staining. Vehicle control showed severe inflammation and infiltration of immune cells into the articular capsule compared to the normal control, Erdr1-treated group, and MTX-treated group (original magnification ×200). **C.** The mean arthritis score was sharply increased in CIA-induced vehicle control, whereas the severity was attenuated by Erdr1 treatment. All values were analyzed using an unpaired Student's *t*-test. Vehicle *versus* rErdr1-treated group, *P < 0.05, **P < 0.001 **D.** Paw thickness was significantly increased in vehicle control until day 31. Compared with vehicle control, thickness was reduced in the Erdr1-treated group as a result of decreased swelling. Vehicle *versus* rErdr1-treated group, *P < 0.05, **P < 0.001 **E.** Anti-CII antibodies in mouse serum were measured by ELISA. The Erdr1-treated group showed decreased level of autoantibody production. *P < 0.05, **P < 0.001, ***P < 0.0001.

To examine direct effects of rErdr1 on arthritic joint inflammation, we used a widely used animal model of RA. DBA/1J mice were immunized and subsequently boosted with CII to induce CIA and then treated with rErdr1 or PBS (vehicle control) three times/week. Compared to negative control, CIA mice subsequently treated with PBS showed severely inflamed paws with redness and swelling, whereas CIA mice treated rErdr1 showed significant reduction of paw inflammation together with decreased redness and swelling (Figure 1A). Histological analysis showed severe inflammation in PBS-treated CIA mice compared to the normal negative controls. Also, infiltration of immune cells into the articular capsule was detected in vehicle control mice. However, infiltration of immune cells was considerably reduced by rErdr1 treatment in PBS-treated CIA mice, and articular capsules were similar in appearance to those of normal controls (Figure 1B). As shown in Figure 1C, PBS-treated CIA mice showed accelerated onset of CIA 20 days after immunization. The arthritis severity score of these mice was sharply increased; however, the score was significantly decreased in rErdr1-treated mice. The maximal difference between PBS- and rErdr1-treated CIA mice was seen 31 days after immunization. In addition, paw thickness measured using a thickness gauge was significantly decreased in the rErdr1-treated compared with the PBS-treated CIA mice (Figure 1D). These data suggest that rErdr1 treatment strongly attenuated the development and progression of CIA.

### rErdr1 suppresses anti-CII antibody levels in the serum of CIA mice

Anti-CII antibodies are significantly increased in patients with RA as rheumatoid factors. These autoantibodies contribute to arthritis through Fc receptor (FcR)-mediated immune cell activation. To determine the levels of anti-collagen antibodies, including total IgG, IgG1, IgG2a, and IgM in the serum of each mouse, ELISA was performed. Levels of anti-collagen antibodies were greatly increased in PBS-treated CIA mice, but were barely detectable in negative control mice. Serum levels of anti-collagen total IgG, IgG1, IgG2a, and IgM were greatly reduced by administration of rErdr1, showing the inhibitory effects of rErdr1 on autoantibody production in the CIA mouse model (Figure 1E). Taken together, these data suggest that rErdr1 has a therapeutic effect on CIA *in vivo*.

### Erdr1 suppresses cell migration through inhibition of ERK1/2 in SW982 synovial fibroblasts

**Figure 2:**
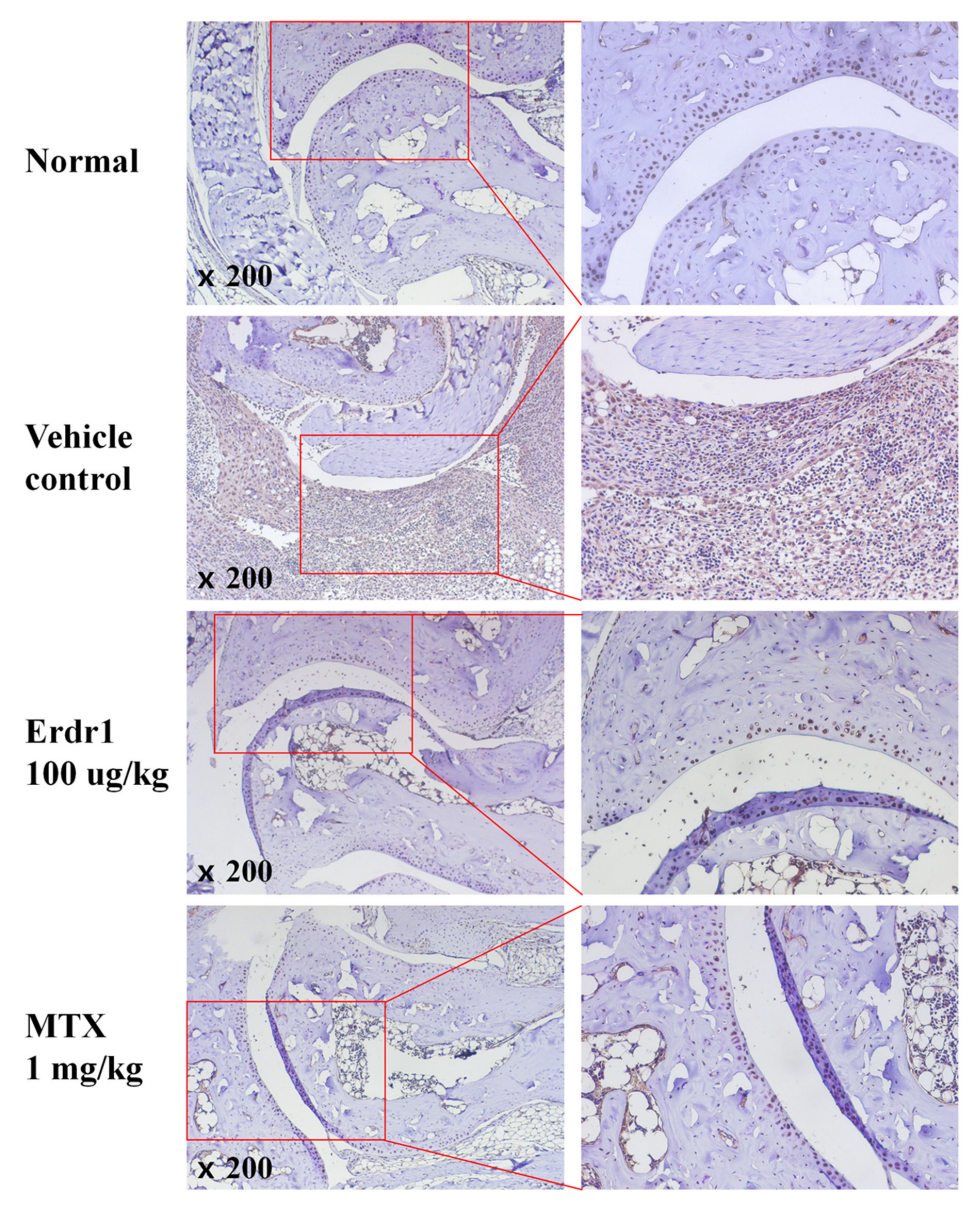
Erdr1 downregulates IL-18 expression in synovial tissue of CIA mice Immunohistochemical analysis was carried out to detect IL-18 expression in synovial tissue. Paraffin sections were prepared and blocked in PBS containing 5% goat serum and 0.1% BSA. Sections were treated with rabbit anti-mouse IL-18 (1:1000 dilution) antibody and HRP-conjugated goat anti-rabbit IgG (1:1000 dilution). Sections were treated with DAB to detect target protein expression and observed under a microscope (original magnification ×200).

It has been reported that IL-18 is elevated in synovial tissue and fluid of RA patients and that it enhances inflammatory responses and angiogenesis in RA joints [5]. Based on our previous finding that Erdr1 expression is negatively correlated with that of IL-18 [6], we investigated whether IL-18 expression in synovial tissue was decreased in the rErdr1-treated group. Figure 2 shows that IL-18 expression was significantly elevated in synovial tissue from vehicle control PBS-treated CIA mice, whereas it was decreased in rErdr1-treated CIA mice as negative control. The elevated IL-18 in RA synovium contributes to arthritis, resulting in severe inflammation, including inflammatory cell infiltration and pannus formation [5].

**Figure 3:**
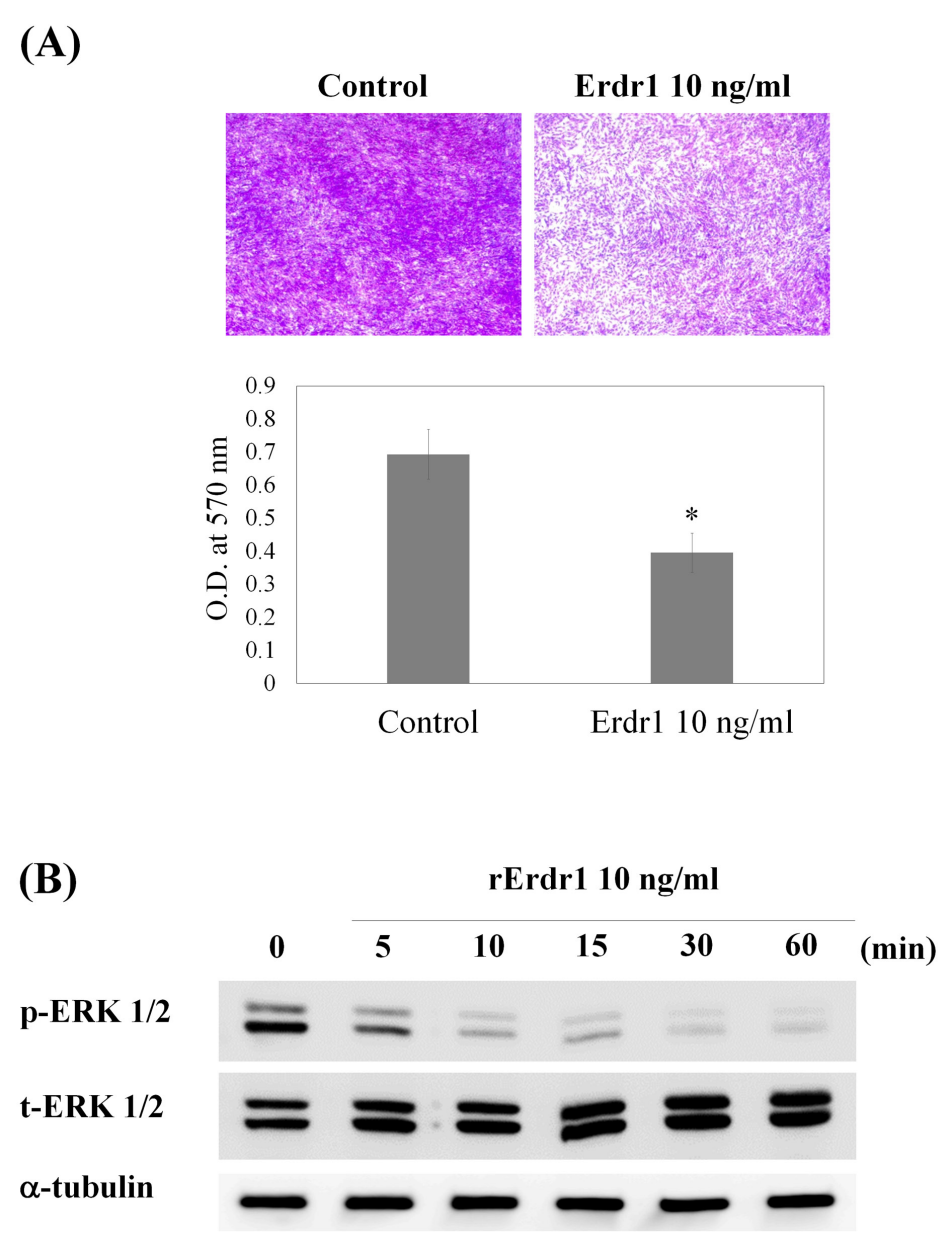
Erdr1 inhibits migration of SW982 synovial fibroblasts by downregulating ERK1/2 **A.** SW982 cells were treated with recombinant Erdr1 (10 ng/ml) for 24 h. Equal numbers of cells were added to the upper insert in serum-free DMEM. Transwell chambers were incubated at 37°C in a 5% CO_2_ humidified incubator for 24 h. Cells that had migrated to the underside of the transwell membrane were then fixed in methanol, stained with crystal violet, and observed under a microscope using a 40× objective. After imaging, the stain was eluted from the cells in 10% acetic acid, and optical density (O.D.) at 570 nm was measured using an ELISA reader. All values were analyzed using an unpaired Student's *t*-test. *P < 0.05 **B.** To identify related signaling pathways, rErdr1-mediated ERK1/2 phosphorylation was tested. Cells were treated with rErdr1 (10 ng/ml) for the indicated times from 5 min to 60 min and then lysed. Western analysis was performed to detect total and phosphorylated ERK1/2.

In addition, increased migration of synovial fibroblasts is strongly related to RA pathogenesis. In RA, synovial fibroblasts migrate to unaffected synovium, resulting in the spread of arthritis and destruction of distant joints [10]. To investigate whether rErdr1 has a therapeutic effect on RA by reducing migration of synovial fibroblasts, the migratory ability of SW982 cells was tested by transwell migration assay. As shown in Figure 3A, Erdr1 significantly decreased migration of SW982 cells. ERK1/2 phosphorylation is associated with increased cell migration, invasion, and proliferation. Therefore, ERK1/2 phosphorylation was examined in Erdr1-treated SW982 cells by western analysis to determine whether this signaling pathway is involved in Erdr1-regulated migration. Figure 3B shows that ERK1/2 phosphorylation was markedly decreased by rErdr1 treatment. These data suggest that rErdr1 exerts a therapeutic effect in CIA mouse model by downregulation of synovial fibroblast migration through inhibition of ERK1/2.

## DISCUSSION

RA is an autoimmune disease that is accompanied by chronic inflammation of the joint. A representative pro-inflammatory cytokine, IL-18, is highly expressed in RA patients, and positive correlations between IL-18 level and disease activity have been demonstrated [11]. Elevated IL-18 activates resident synovial cells as well as immune cells. In the RA joint, IL-18 activates RA synovial fibroblasts to produce RANKL, M-CSF, GM-CSF, and osteoprotegerin (OPG), which are known osteoclast differentiation factors, resulting in increased bone destruction by osteoclasts [12]. In addition, IL-18 exacerbates RA pathogenesis through severe joint inflammation by increasing chemotaxis of multiple immune cells. IL-18 enhances expression of adhesion molecules, such as ICAM-1 and VCAM-1, which contribute to cell migration, and acts as a chemoattractant for CD4+ T cells in synovium from RA patients [13]. Also, IL-18 stimulates chemokines, including CCL20, and angiogenic factors, including VEGF, resulting in increased chemotaxis of monocytes and neutrophils and angiogenesis in the affected RA synovium [14]. Therefore, inhibition of IL-18 expression and activity is a potential target for RA therapy.

In our previous studies, we determined that Erdr1 expression is increased by siRNA-mediated IL-18 knockdown in a human keratinocyte cell line. Also, there is a significant negative correlation between the level of IL-18 and Erdr1 expression in lesional skin of psoriasis patients [6], implying opposing effects of IL-18 and Erdr1. Based on this negative correlation, we hypothesized that Erdr1 may exert anti-inflammatory effects, in contrast to the pro-inflammatory effect of IL-18. Because it is well known that IL-18 expression is positively correlated with severity and progression of RA, we investigated the anti-arthritic effects of Erdr1 using the CIA mouse model. In this study, we determined that Erdr1 has a significant therapeutic effect on RA. Arthritis severity score, histological features, and serum levels of autoantibodies are reduced by rErdr1 treatment. Also, IL-18 expression in the affected synovium is markedly decreased in the Erdr1-treated group of CIA mice. In fact, attempts have been made to develop therapeutic approaches that incorporate factors such as caspase-1 inhibitors, IL-18-binding protein, and neutralizing antibodies to inhibit IL-18 expression and activity.

RA is characterized by hyperplasia of synovial fibroblasts in the affected synovium. This hyperproliferation results in the formation of an RA pannus, which erodes surrounding bone. Also, synovial fibroblasts migrate to unaffected synovium in RA patients, and then drive systemic inflammatory processes [10]. During the inflammatory process, synovial fibroblasts produce MMPs that break down cartilage, and RANKL, which promotes osteoclast differentiation, resulting in bone erosion. Various inflammatory mediators, including cytokines and chemokines, secreted by synovial fibroblasts also exacerbate inflammation in RA synovium. The released inflammatory mediators stimulate the production of other inflammatory mediators and promote the infiltration of immune cells, thus promoting and sustaining inflammation in the synovium [15]. Consequently, synovial fibroblasts are good targets for anti-inflammatory drugs. Here, we determined the anti-migratory activity of Erdr1 in synovial fibroblasts. In addition, our previous study suggests a pro-apoptotic role of Erdr1 in mouse melanoma [8], implying that Erdr1 might act as a pro-apoptotic factor in synovial fibroblasts to inhibit hyperproliferation.

In conclusion, this study determined that rErdr1 has a therapeutic effect on RA in a mouse model of CIA. Erdr1 suppresses IL-18 expression in synovial tissue and inhibits synovial fibroblast migration through inhibition of ERK1/2 phosphorylation, indicating that Erdr1 might be part of an effective therapeutic approach for RA based on its anti-inflammatory properties.

## MATERIALS AND METHODS

### Mouse model of CIA

Male 6-week old DBA/1J mice (Central Lab. Animal Inc., Seoul, Korea) were used to induce CIA. Bovine type II collagen (CII, 2 mg/ml; Chondrex, Redmond, WA, USA) was diluted in an equal volume of complete Freund's adjuvant (Chondrex). Mice were first immunized with 50 μl emulsion containing 50 μg CII *via* intradermal (i.d.) injection into the tail. After 14 days, a booster injection of 50 μl of an emulsion of CII in incomplete Freund's adjuvant (Chondrex) was administered through i.d. injection at the same site in the tail. After booster, Erdr1 (100 μg/kg) or phosphate-buffered saline (PBS) (vehicle control) was administered by intraperitoneal (i.p.) injection three times/week. All experimental procedures involving mice were approved by the Institutional Animal Care and Use Committee of Sookmyung Women's University.

### Evaluation of arthritis severity

The severity of paw inflammation was evaluated on a scale of 0 to 4 (0, normal; 1, mild symptoms confined to the tarsals or ankle joint; 2, mild symptoms extending from the ankle to the tarsals; 3, moderate symptoms; 4, severe symptoms). Inflammation was scored by researchers blinded to the identities of the animal groups. The final score was the sum of the overall scores for each paw. To further confirm paw inflammation, paw thickness was measured using a Mitutoyo loop handle dial thickness gauge with a round disc.

### Measurement of anti-collagen Ig titers

Blood samples were collected from the tails of mice on day 28 after the first immunization with CII, and serum was collected after clotting at room temperature for 30 min. To measure anti-collagen immunoglobulins IgG, IgG1, IgG2a, and IgM, enzyme-linked immunosorbent assay (ELISA) was performed. Briefly, 96-well plates (Nunc) were coated with 4 μg/ml CII in 0.05 M sodium carbonate at 4°C. After washing with Tris-buffered saline containing 0.05% Tween 20 (TBST), nonspecific binding was blocked by incubation of wells with 200 μl 1% BSA (Sigma) in TBS at room temperature for 30 min. After washing, 100 μl serum diluent (1:25,000 for IgG; 1:12,500 for IgG1, IgG2a, and IgM) were applied and incubated at room temperature for 1 h. Diluted HRP-conjugated goat anti-mouse IgG, IgG1, IgG2a, and IgM (Santa Cruz Biotechnology, Santa Cruz, CA, USA) were added to wells followed by incubation at room temperature for 1 h. Wells were washed, and 100 μl HRP liquid substrate tetramethylbenzidine (TMB) (Sigma) were added and incubated in darkness. After addition of 50 μl stop reagent for TMB (Sigma), absorbance was measured at 450 nm.

### Immunohistochemistry

For histological evaluation of the joints, forelimbs and hindlimbs from each mouse were fixed in 4% paraformaldehyde overnight. Fixed tissues were decalcified and embedded in paraffin, and 8-μm sections were prepared. Sections were stained with hematoxylin and eosin (H&E) to visualize nuclei and cytoplasm. Sections were mounted and observed on a microscope using a 100× objective. For examination of IL-18 expression in synovial tissues, sections were blocked in 5% goat serum and 0.1% bovine serum albumin (BSA) in PBS followed by treatment with rabbit anti-mouse IL-18 (1:1000 dilution) antibody and horseradish peroxidase (HRP)-conjugated goat anti-rabbit IgG (1:1000 dilution). Goat IgG was used as isotype control. Sections were treated with HRP substrate 3,3′-diaminobenzidine (DAB) and observed under a microscope.

### Transwell migration assay

Migration assays were performed using 24-well Transwell^®^ culture chambers (Costar, Cambridge, MA). Lower chambers were filled with Dulbecco's modified Eagle medium (DMEM) containing 10% fetal bovine serum (FBS). Equal numbers (3×10^4^) of SW982 human synovial fibroblasts were added to the upper insert in serum-free DMEM. Transwell chambers were incubated at 37°C in a 5% CO_2_ humidified incubator for 24 h. After incubation, cells that had migrated to the underside of the membrane were fixed in methanol and stained with crystal violet. After imaging, stain was eluted from the cells in 10% acetic acid, and optical density (O.D.) at 570 nm was measured using an ELISA reader (Molecular Devices, Sunnyvale, CA, USA). The migratory ability of cells was assessed in triplicate wells.

### Western blotting

SW982 cells were washed with cold PBS, and proteins were extracted using the ProPrep Protein Extraction Kit containing five protease inhibitors (Intron, Seongnam, Korea) at 4°C for 30 min. Protein concentration in cell lysates was measured using the Bio-Rad Bradford assay (Hercules, CA, USA). Equal volumes of denatured protein were separated by SDS-PAGE electrophoresis through a 12% gel and transferred to a polyvinylidene fluoride (PVDF) membrane (Bio-Rad). After blocking in PBS containing 5% nonfat dry milk and 0.1% Tween 20 (PBST) for 1 h, mouse anti-phospho-Erk1/2, anti-Erk1/2, or anti-α-tubulin antibody was applied to the membrane and incubated at 4°C overnight. The membrane was washed with PBST and incubated with HRP-conjugated goat anti-mouse IgG (Jackson ImmunoResearch, West Grove, PA, USA) at room temperature for 2 h. Proteins were detected using an Amersham ECL system (GE Healthcare, Buckinghamshire, UK) and LAS-3000 imaging system (Fujifilm).

### Statistical analysis

All values were analyzed with an unpaired Student's *t*-test. Statistical analyses were performed using GraphPad Prism 5 (GraphPad Software, La Jolla, CA, USA). *P* values < 0.05 were considered statistically significant in a two-tailed *t*-test.
